# Knockdown of lncRNA PVT1 inhibits prostate cancer progression in vitro and in vivo by the suppression of KIF23 through stimulating miR-15a-5p

**DOI:** 10.1186/s12935-020-01363-z

**Published:** 2020-07-02

**Authors:** Huijuan Wu, Xin Tian, Chaoyang Zhu

**Affiliations:** 1 Department of Telemedicine and Internet Medical Center, The Huaihe Hospital of Henan University, No. 115 Ximen Avenue, Kaifeng, 475000 Henan China; 2 Department of Urology Surgery, The Huaihe Hospital of Henan University, Kaifeng, Henan China

**Keywords:** PVT1, miR-15a-5p, KIF23, Prostate cancer

## Abstract

**Background:**

Prostate cancer (PCa) greatly threatens men’s lives, with high incidence and mortality. Recently, the research of long non-coding RNAs (lncRNAs) has made breakthroughs in the development of human cancers. This study aimed to figure out the role and action mechanism of lncRNA PVT1 (PVT1) in PCa.

**Methods:**

The expression of PVT1, microRNA-15a-5p (miR-15a-5p) and kinesin family member 23 (KIF23) was detected by quantitative real-time polymerase chain reaction (qRT-PCR). Cell proliferation, apoptosis, migration and invasion were assessed by 3-(4,5-dimethyl-2-thiazolyl)-2,5-diphenyl-2-*H*-tetrazolium bromide (MTT), flow cytometry and transwell assays, respectively. The protein levels of KIF23 and proliferation, apoptosis, and epithelial-mesenchymal transition (EMT)-related markers were quantified by western blot. The relationship between miR-15a-5p and PVT1 or KIF23 was predicted by starBase v2.0 and verified by dual-luciferase reporter assay. Xenograft assay was conducted to determine the role of PVT1 in vivo.

**Results:**

The expression of PVT1 and KIF23 was enhanced, while miR-15a-5p expression was reduced in PCa tissues and cells. PVT1 interference inhibited proliferation, migration and invasion but promoted apoptosis of PCa cells. MiR-15a-5p was a target of PVT1, and KIF23 was a target of miR-15a-5p. The inhibition of miR-15a-5p reversed the effects of PVT1 interference and suppressed the roles of KIF23 knockdown. KIF23 expression was regulated by PVT1 through miR-15a-5p. PVT1 interference blocked PCa progression in vivo.

**Conclusion:**

PVT1 knockdown had effects on the progression of PCa by inhibiting the expression of KIF23 via enriching miR-15a-5p in vitro and in vivo, suggesting that PVT1 might be a novel biomarker for the treatment of PCa.

## Highlights

1. PVT1 and KIF23 are up-regulated, while miR-15a-5p is down-regulated in PCa tissues and cells.
2. PVT1 interference attenuates malignant activities of PCa cells in vitro.
3. PVT1 directly targets miR-15a-5p, and miR-15a-5p directly binds to KIF23.
4. PVT1 interference regulates PCa progression by the regulation of KIF23 and miR-15a-5p.
5. PVT1 knockdown impedes PCa development in vivo.

## Background

Prostate cancer (PCa), a common malignancy in the male population, is an important cause of male mortality [1]. The occurrence of PCa is more prevalent in developed countries [2, 3]. The complex mechanism of PCa initiation and development involves various factors, such as age, lifestyle, environment, and heredity [1, 4]. Androgen deprivation therapy is the mainstream method for the treatment of PCa and has a considerable curative effect in the early stage [5, 6]. In general, inappropriate screening schedules, low cure rates, and drug resistance are substantial burdens in the treatment of PCa [1]. Therefore, the underlying mechanisms associated with the development and progression of PCa and novel targeted specific biomarkers still require further investigation.

Long non-coding RNAs (lncRNAs), over 200 nucleotides in length, are a kind of RNA molecules with extensive functionality [7]. Although our current understanding of the role of lncRNAs is limited, existing reports reveal partial functions of lncRNAs involved in nuclear structural integrity, modulation of gene expression, chromatin remodeling, transcription and post-transcriptional processing [8]. Recently, the involvement of lncRNAs in cancer progression has attracted much attention [9]. In PCa, dozens of lncRNAs have been identified as biologically significant. For example, lncRNA SNHG20, with a high level in PCa tissues and cells, contributed to the proliferation and invasion of PCa cells [10]. LncRNA MALAT1 was also overexpressed in PCa, and its high expression was associated with tumor stage, drug resistance, tumorigenicity and progression of PCa [11]. LncRNA Plasmacytoma Variant Translocation 1 (PVT1), was well known to play a part in tumorigenesis in multiple cancer types [12–14]. In this study, a novel mechanism of PVT1 involving in the development of PCa was identified.

MicroRNAs (miRNAs), 18–24 nucleotides in length, generally play biological functions by acting as the downstream target of lncRNAs [15, 16]. As a kind of non-coding RNAs, the functional roles of miRNAs have been well investigated. In cancer, miRNAs serve as tumor suppressors or promoters by competitively modulating the expression of downstream specific target genes [16, 17]. Existing reports stated that miR-15a-5p participated in numerous cancers, such as breast cancer [18], papillary thyroid cancer [19], and endometrial cancer [20]. However, the function of miR-15a-5p in PCa was not fully elucidated, and related mechanism of miR-15a-5p in PCa was lacking. Kinesin family member 23 (KIF23) locates at the interzone of mitotic spindles and functions by driving microtubule movement [21]. Previous studies demonstrated that KIF23 had been identified as a novel therapeutic target in the treatment of lung cancer, pleural mesothelioma, and gastric cancer [22–24]. Unfortunately, the function of KIF23 in the progression of PCa was limited to mention yet. Accordingly, our study devoted to exploring the potential roles of miR-15a-5p and KIF23 in the tumorigenesis and progression of PCa.

Here, we investigated the expression of PVT1 in PCa tissues and cell lines, as wells as the expression of miR-15a-5p and KIF23. The specific function of PVT1 was determined in vitro and in vivo. The relationship between miR-15a-5p and PVT1 or KIF23 was confirmed, so as to provide a new sight and a novel mechanism for the progression of PCa.

## Materials and methods

### Specimens

This research acquired the approval of the Ethics Committee of the Huaihe Hospital of Henan University. A total of 25 paired PCa tissues and adjacent normal tissues were collected from the Huaihe Hospital of Henan University. After removal, all specimens were quickly placed into liquid nitrogen and stored at − 80 °C soon after. Each subject had signed the informed consent prior to the surgery. The clinicopathological characteristics of PCa patients were recorded in Additional file 1: Table S1.

### Cell lines and cell culture

PCa cell lines (22RV1 and DU145), normal prostate epithelial cell line (RWPE-1) and embryonic kidney cells (293T) were all purchased from BeNa Culture Collection (Suzhou, China). According to the instruction, 22RV1 cells were maintained in 90% Roswell Park Memorial Institute 1640 (RPMI 1640; Sigma, St. Louis, MO, USA) containing 10% fetal bovine serum (FBS; Sigma). DU145, RWPE-1 and 293T cells were cultured in 90% Dulbecco’s Modified Eagle Medium (DMEM; Sigma) containing 10% FBS (Sigma). These cell lines were maintained at 37 °C conditions containing 5% CO_2_.

### Quantitative real-time polymerase chain reaction (qRT-PCR)

MiRNeasy kit (Qiagen, Hilden, Germany) was used for RNA extraction. For PVT1 and KIF23, OneStep RT-PCR Kit (Qiagen) was adapted for reverse transcription (RT) reactions to assemble complementary DNA (cDNA). For miR-15a-5p, miScript II RT Kit (Qiagen) was used for synthesizing cDNA. QuantiFast SYBR Green PCR Kit (Qiagen) was used to perform qRT-PCR using the Step One Plus real-time PCR system (Applied Biosystems, Foster City, CA, USA). Relative expression was normalized by β-actin or small nuclear RNA U6 and calculated using the 2^−∆∆Ct^ method. The primers were displayed as below: PVT1: 5′- CAGCACTCTGGACGGAC-3′ (forward) and 5′- CAACAGGAGAAGCAAACA-3′ (reverse); KIF23: 5′-AGACAGAAGGCGAGGGATG-3′ (forward) and 5′- GGAGACGAATTGGTGGTGC-3′ (reverse); miR-15a-5p: 5′- TAGCAGCACATAATGGTTTGT-3′ (forward) and 5′- GCGAGCACAGAATTAATACGAC-3′ (reverse); β-actin: 5′-TGGCACCCAGCACAATGAA-3′ (forward) and 5′-CTAAGTCATAGTCCGCCTAGAAGCA-3′ (reverse); U6: 5′- CAGCACATATACTAAAATTGGAACG-3′ (forward) and 5′- ACGAATTTGCGTGTCATCC-3′ (reverse).

### Cell transfection

For PVT1 downregulation, small interference RNA (siRNA) against PVT1 (si-PVT1) and its negative control (si-NC) were assembled by Sangon Biotech (Shanghai, China). For stable PVT1 knockdown, lentiviral vector (lenti-short hairpin sh-PVT1) and its negative control (sh-NC) were obtained from Genechem (Shanghai, China). For miR-15a-5p inhibition or enrichment, miR-15a-5p inhibitor (5′-CACAAACCAUUAUGUGCUGCUA-3′) or miR-15a-5p mimic (sense: 5′-UAGCAGCACAUAAUGGUUUGUG-3′ and antisense: 5′-CAAACCAUUAUGUGCUGCUAUU-3′) and negative control (inhibitor NC (5′-CAGUACUUUUGUGUAGUACAA-3′) or miR-NC (sense: 5′-UUCUCCGAACGUGUCACGUTT-3′ and antisense: 5′-ACGUGACACGUUCGGAGAATT-3′)) were purchased from Ribobio (Guangzhou, China). For KIF23 knockdown, siRNA against KIF23 (si-KIF23) and si-NC were also constructed by Sangon Biotech. The 22RV1 and DU145 cells were subjected to transfection with above items using Lipofectamine 2000 (Invitrogen, Carlsbad, CA, USA). Following experiments were conducted at 48 h post-transfection.

### 3-(4,5-Dimethyl-2-thiazolyl)-2,5-diphenyl-2-*H*-tetrazolium bromide (MTT) assay

22RV1 and DU145 cells with different transfection were seeded into 96-well plates (Corning Costar, Corning, NY, USA) at a density of 5 × 10^3^ cells per well. Then 10 µL MTT solution (Beyotime, Shanghai, China) was pipetted into each well at 0 h, 24 h, 48 h and 72 h for another 4 h at 37 °C. After that, the dimethyl sulfoxide (DMSO; Beyotime) was added into each well to dissolve the formazan. The absorbance was measured at 490 nm using the Multiskan Ascent (Thermo Fisher Scientific, Waltham, MA, USA) to assess cell proliferation.

### Flow cytometry assay

22RV1 and DU145 cells with different transfection were seeded into 6-well plates. Then, 0.25% trypsin was used to deal with the cells. Next, the cells after washing with phosphate buffer saline (PBS) were detected by Annexin V-FITC Apoptosis Detection Kit (KeyGEN, Nanjing, China). In brief, cells were resuspended by 500 µL binding buffer and then incubated by 5 µL Annexin V-fluorescein isothiocyanate (FITC) and propidium iodide (PI) for 15 min without light. Finally, the apoptotic cells were analyzed using flow cytometer (BD Biosciences, San Jose, CA, USA).

### Transwell assay

The abilities of migration and invasion were assessed using 6-well transwell chambers (Corning Costar). Briefly, cells (1 × 10^5^ cells/mL) were resuspended in fresh DMEM or RPMI1640 containing 10% FBS. Then, the suspensions were pipetted into the upper chambers, and DMEM or RPMI1640 containing 10% FBS was added into the lower chambers. After culturing for 24 h, the migrated cells in the low surface were fixed with formaldehyde and stained with 5% crystal violet. The cells were counted in five random fields using a microscope (Olympus, Tokyo, Japan) with magnification of 100×. Noteworthily, the ability of cell invasion was also assessed using the same method, except that the upper transwell chambers needed to be coated with Matrigel (Corning Costar) before the inoculation.

### Western blot

Western blot analysis was executed based on the methods described previously [25]. The primary antibodies against Vimentin (ab193555; 1:1000; Abcam, Cambridge, MA. USA), E-Cadherin (E-cad) (ab133597; 1:1000; Abcam), Caspase-3 (ab13847; 1:1000; Abcam), CyclinD1 (ab16663; 1:1000; Abcam), KIF23 (PA5-31773; 1:1000; Invitrogen) and β-actin (ab8227; 1:5000; Abcam) were used in this study, and goat anti-rabbit antobodies (ab205718; 1:5000; Abcam) were acted as the second antibodies. The protein blots were quantified using the Image J software (version 1.46; National Institutes of Health, Bethesda, MA, USA).

### Bioinformatics analysis and dual-luciferase reporter assay

The online tool starBase v2.0 (http://starbase.sysu.edu.cn/) was used to predict the potential target genes and analyze the specific binding sites.

The relationship between miR-15a-5p and PVT1 or KIF23 was verified by dual-reporter assay. In brief, the fusion plasmids (pRL-CMV, Promega, Madison, WI, USA) containing the sequences of PVT1 wild type (WT-PVT1) with the miR-15a-5p binding sites or PVT1 mutant (MUT-PVT1) with the mutated miR-15a-5p binding sites, or containing the sequences of wild type KIF23 3′ untranslated region (UTR) (KIF23 3′ UTR-WT) with the miR-15a-5p binding sites or mutant KIF23 3′ UTR (KIF23 3′ UTR-MUT) with the mutated miR-15a-5p binding sites were obtained from Sangon Biotech. The 293T cells were cotransfected with above fusion plasmids and miR-15a-5p or miR-NC, respectively. After 48 h-transfection, the luciferase activity was determined using the Dual-Luciferase Reporter Assay Kit (Promega).

### Xenograft assay

All animal procedures were approved by the Animal Care and Use Committee of the Huaihe Hospital of Henan University. A total of 12 BALB/c nude mice (6-week-old, male) were purchased from HFK bioscience Co., LTD (Beijing, China). DU145 cells packaged with lentiviral vector containing sh-PVT1 or sh-NC were planted into 24-well plates (5 × 10^4^ cells/well). Then, different concentrations of puromycin (1, 2, 5, 10 and 15 µg/mL) were added into different wells, and the cell viability was monitored every day to screen stable cells with transfection. DU145 cells with stable sh-PVT1 or sh-NC transfection were subcutaneously injected into the right flank of mice (n = 6 per group). Ten days after inoculation, the tumor volume was measured every 4 days based on the formula: length× width^2^ × 0.5. Afterwards, the mice were killed after 34 days, and the tumor tissues were collected for the following analyses.

### Statistical analysis

The data were analyzed by GraphPad Prism 5.01 (GraphPad Software, Inc., La Jolla, CA, USA) and presented as the mean ± standard deviation (SD). Each experiment was repeated at least 3 times. The differences were distinguished by Student’s *t*-test or one-way analysis of variance (ANOVA). The correlation analysis was performed according to the Spearman’s correlation coefficient. *P *< 0.05 was considered to be a statistically significant difference.

## Result

### PVT1 was up-regulated in PCa tissues and cells

**Fig. 1:**
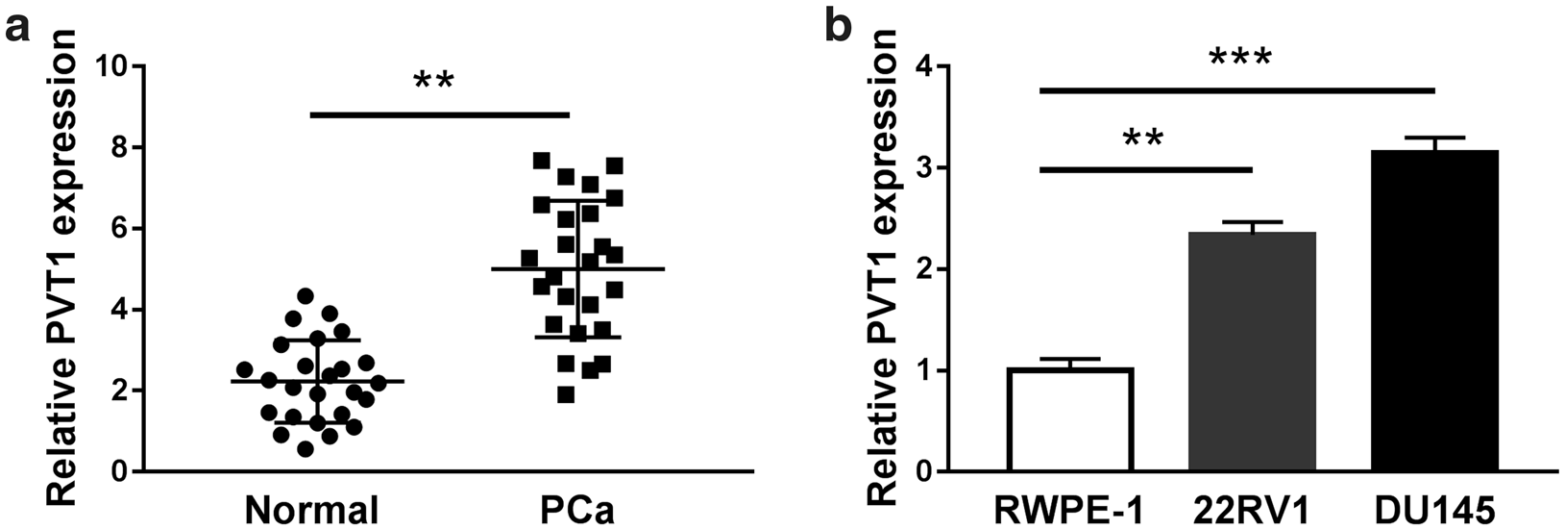
The expression of PVT1 was enhanced in PCa tissues and cell lines. **a** The expression of PVT1 in PCa tissues (n = 25) and adjacent normal tissues (n = 25) was detected by qRT-PCR. **b** The expression of PVT1 in 22RV1, DU145 and RWPE-1 cells was also detected by qRT-PCR. **P < 0.01, ***P < 0.001

To define the expression of PVT1 in PCa, qRT-PCR was carried out. The result showed that PVT1 was highly expressed in PCa tissues (n = 25) compared with that in normal tissues (n = 25) (Fig. 1a). Likewise, the expression of PVT1 in PCa cell lines (22RV1 and DU145) was higher than that in a normal prostate epithelial cell line (RWPE-1) (Fig. 1b). The data indicated that PVT1 was aberrantly regulated in PCa and might play a part in the development of PCa.

### Interference of PVT1 inhibited proliferation, migration and invasion but induced apoptosis of PCa cells

**Fig. 2:**
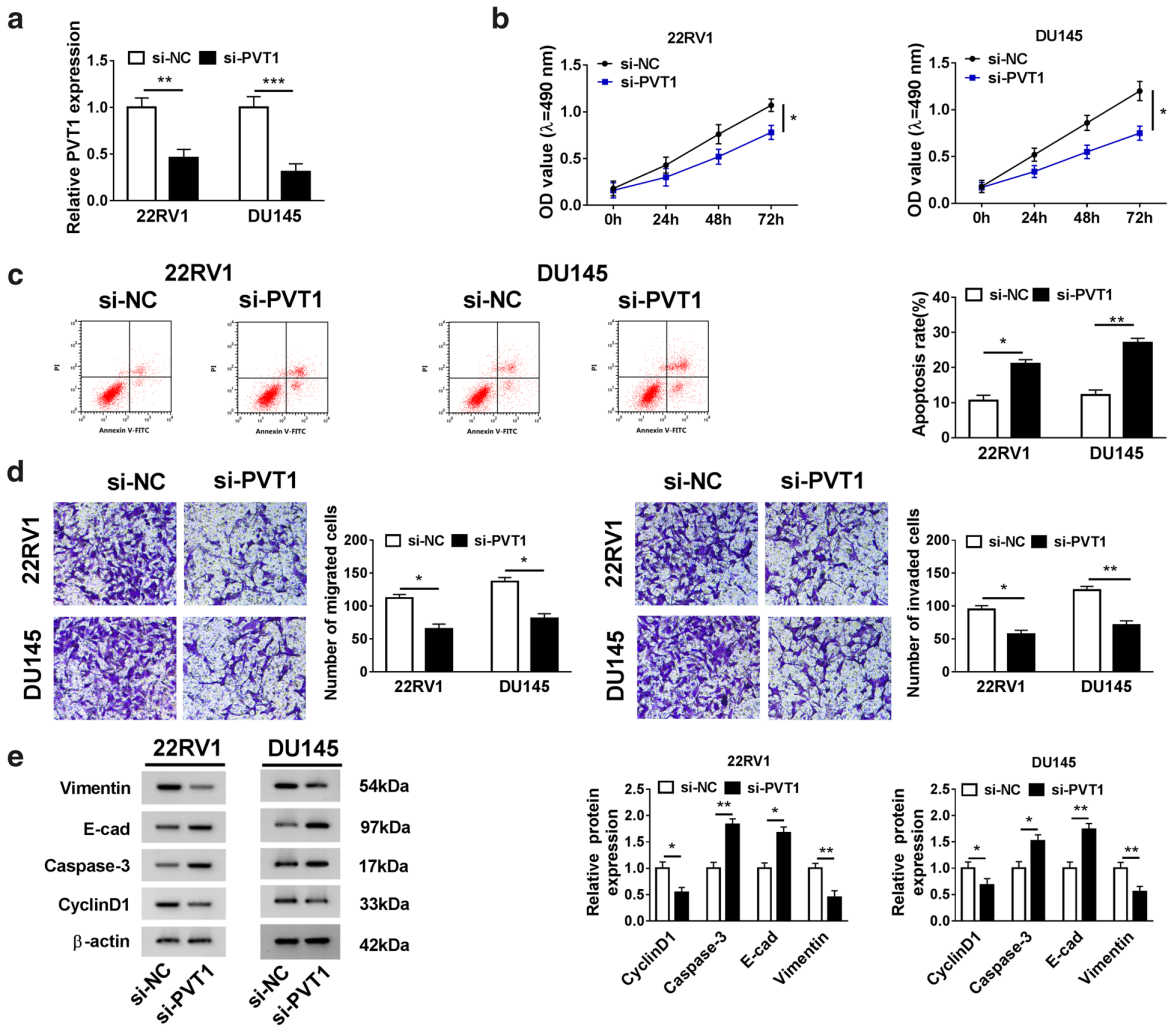
Downregulation of PVT1 inhibited proliferation, migration and invasion but promoted apoptosis of PCa cells. **a** The efficiency of PVT1 interference was examined by qRT-PCR. **b** Cell proliferation was assessed using MTT assay. **c** Cell apoptosis was monitored by flow cytometry. **d** Cell migration and invasion were characterized by transwell assay (×100). **e** The levels of Vimentin, E-cad, Caspase-3 and CyclinD1 were quantified by western blot. *P < 0.05, **P < 0.01, ***P < 0.001

To determine the role of PVT1 on cell proliferation, apoptosis, migration and invasion, the endogenous level of PVT1 was knocked down in 22RV1 and DU145 cells. The efficiency of PVT1 interference was checked using qRT-PCR, and the data displayed that PVT1 was noticeably down-regulated in PCa cells transfected with si-PVT1 (Fig. 2a). The proliferation of 22RV1 and DU145 cells was significantly repressed in response to PVT1 interference by MTT assay (Fig. 2b). Flow cytometry assay presented that interference of PVT1 notably increased the apoptosis rate of 22RV1 and DU145 cells compared with sh-NC (Fig. 2c). In addition, transwell assay was conducted to determine the effects on cell invasion and migration. The data presented that interference of PVT1 notably reduced the number of migrated and invaded cells (Fig. 2d). Additionally, several protein markers of proliferation, apoptosis, migration and invasion were quantified, and the result suggested that the levels of Vimentin and CyclinD1 were prominently decreased, while the levels of Caspase-3 and E-cad were pronouncedly increased in 22RV1 and DU145 cells transfected with si-PVT1 (Fig. 2e). Abovementioned analyses suggested that PVT1 knockdown suppressed the malignant phenotypes of PCa cells.

### MiR-15a-5p was targeted by PVT1, and its inhibition reversed the effects of PVT1 interference in PCa cells

**Fig. 3:**
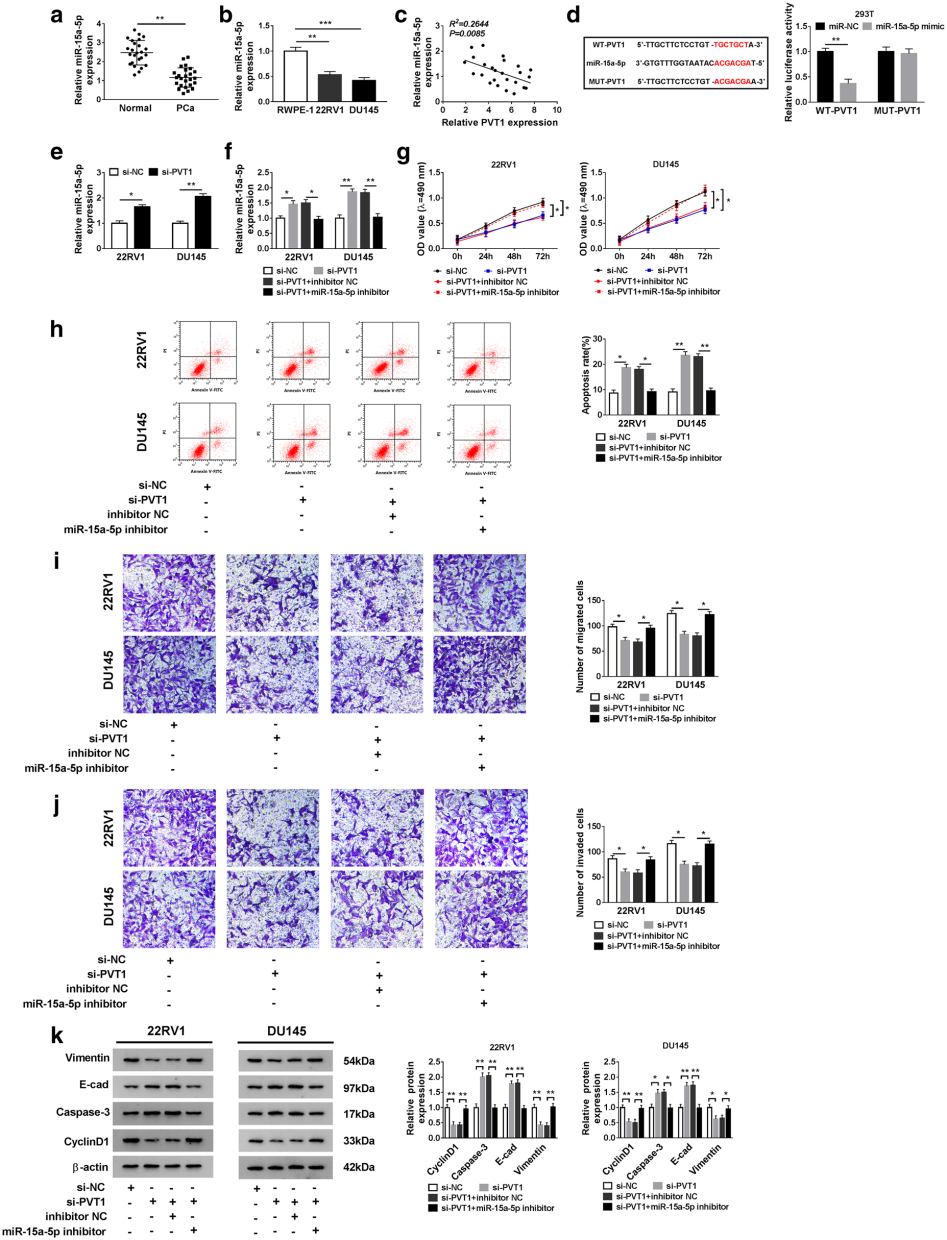
MiR-15a-5p was a target of PVT1, and miR-15a-5p inhibition reversed the effects of PVT1 interference in PCa cells. **a**, **b** The expression of miR-15a-5p was measured in PCa tissues and cell lines by qRT-PCR. **c** The correlation between miR-15a-5p expression and PVT1 expression was analyzed according to Spearman’s correlation coefficient. **d** The binding sites between PVT1 and miR-15a-5p were predicted by starBase v2.0, and their relationship was verified by dual-luciferase reporter assay. **e** The expression of miR-15a-5p was affected by the change of PVT1 expression. **f** The expression of miR-15a-5p, **g** cell proliferation, **h** apoptosis, **i** migration (×100), **j** invasion (×100) and **k** the levels of Vimentin, E-cad, Caspase-3 and CyclinD1 were determined in PCa cells transfected with si-PVT1, si-NC, si-PVT1 + miR-15a-5p inhibitor and si-PVT1 + inhibitor NC, respectively. *P < 0.05, **P < 0.01, ***P < 0.001

To explore the potential mechanism of PVT1 in the development of PCa, the potential target miRNAs of PVT1 were predicted by starBase v2.0, including miR-515-5p, miR-24-3p, miR-512-3p, miR-15a-5p, miR-21-5p and miR-17-5p. Compared with other miRNAs, the expression of miR-15a-5p decreased more in PCa tissues, and miR-15a-5p expression increased more in PCa cells transfected with si-PVT1 (Additional file 2: Figure S1). Herein, miR-15a-5p was selected for further analyses. The expression of miR-15a-5p was strongly declined in PCa tissues and cell lines relative to normal tissues and cell line, respectively (Fig. 3a, b). In addition, we found that the expression of miR-15a-5p was negatively correlated to PVT1 expression in PCa tissues (Fig. 3c). According to the predicted binding site between miR-15a-5p and PVT1, the mutant sequence fragment of PVT1 harboring mutated binding site was designed (Fig. 3d). Meanwhile, dual-luciferase reporter assay manifested that the luciferase activity was obviously diminished in 293T cells transfected with miR-15a-5p and WT-PVT1 compared with that in 293T cells transfected with WT-PVT1 and miR-NC, while the luciferase activity did not change with the transfection of miR-15a-5p and MUT-PVT1 (Fig. 3d). In order to verify the influence of PVT1 on miR-15a-5p expression, the expression of miR-15a-5p in 22RV1 and DU145 cells transfected with si-PVT1 or si-NC was observed by qRT-PCR. As shown in Fig. 3e, cells with si-PVT1 transfection showed significantly increased the expression of miR-15a-5p. Afterwards, the miR-15a-5p inhibitor was used to perform rescue experiments. The result of qRT-PCR appeared that the increased expression of miR-15a-5p induced by si-PVT1 was strikingly debilitated in cells with the cotransfection of miR-15a-5p inhibitor (Fig. 3f). MTT assay showed that si-PVT1-inhibited proliferation was recovered by miR-15a-5p inhibition (Fig. 3g). Flow cytometry assay pointed out that si-PVT1-induced apoptosis was suppressed by miR-15a-5p inhibition (Fig. 3h). Transwell assay maintained that the number of migrated and invaded cells was blocked by si-PVT1 but restored by si-PVT1 + miR-15a-5p inhibitor (Fig. 3i, j). Additionally, western blot showed that the levels of Vimentin and CyclinD1 inhibited by si-PVT1 were regained by si-PVT1 + miR-15a-5p inhibitor, while the levels of E-cad and Caspase-3 promoted by si-PVT1 were restrained by si-PVT1 + miR-15a-5p inhibitor (Fig. 3k). These findings hinted that miR-15a-5p was a direct target of PVT1, and miR-15a-5p inhibition could recover the malignant phenotypes of PCa cells suppressed by PVT1 interference.

### KIF23 was a target of miR-15a-5p, and miR-15a-5p regulated its expression

**Fig. 4:**
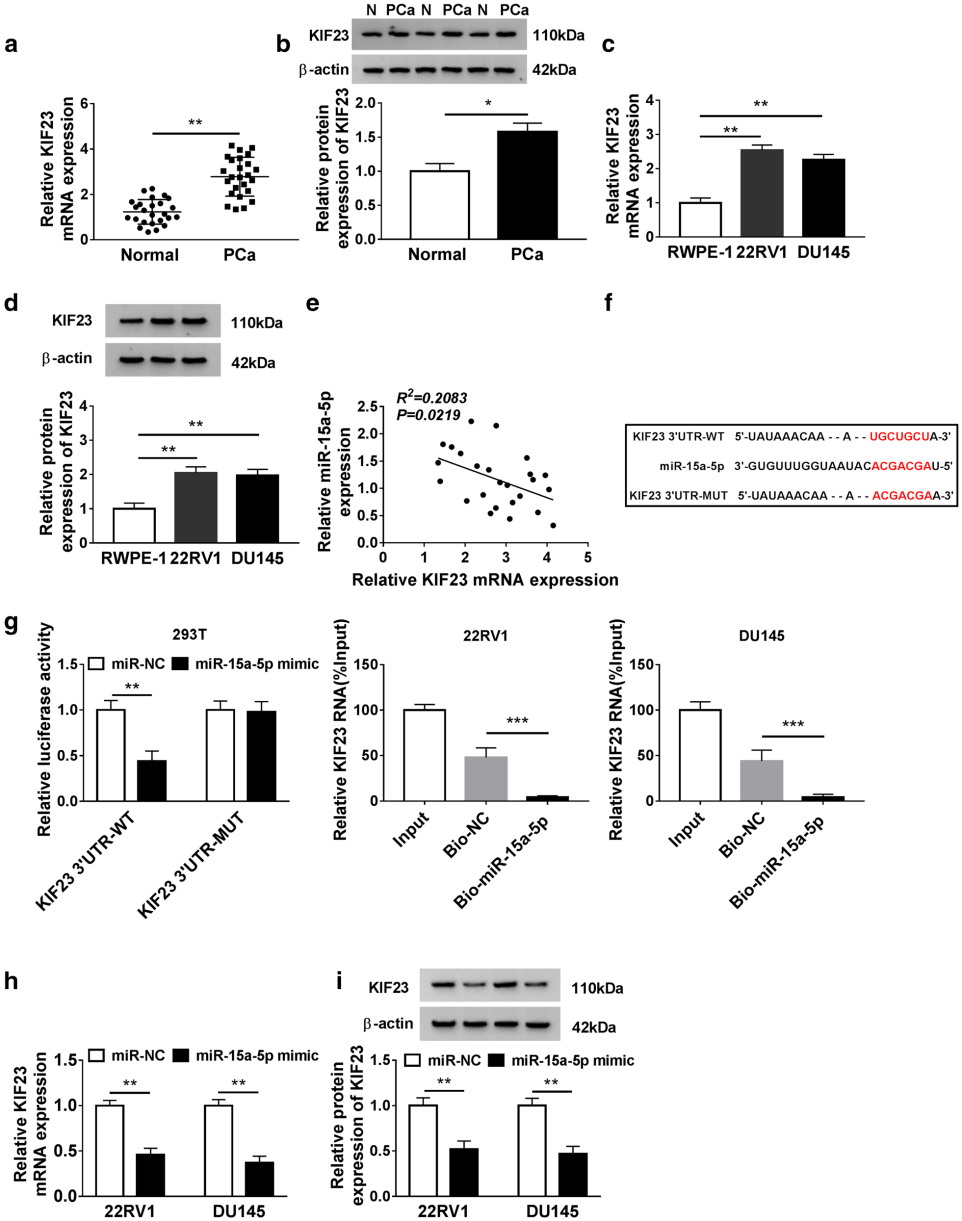
KIF23 was up-regulated in PCa tissues and cell lines and targeted by miR-15a-5p. **a**, **b** The expression of KIF23 at mRNA and protein levels in PCa tissues and normal tissues was measured by qRT-PCR and western blot, respectively. **c**, **d** The expression of KIF23 at mRNA and protein levels in PCa cells and normal cells was measured by qRT-PCR and western blot, respectively. **e** The expression of miR-15a-5p was negatively correlated with KIF23 expression. **f** The binding sites between miR-15a-5p and KIF23 3′ UTR were forecasted by starBase v2.0. **g** The interaction between miR-15a-5p and KIF23 was confirmed by dual-luciferase reporter assay. **h**, **i** The expression of KIF23 was modulated by miR-15a-5p expression at both mRNA and protein levels. *P < 0.05, **P < 0.01, ***P < 0.001

To further explore the action mechanism of PVT1 in the development of PCa, the underlying target mRNAs of miR-15a-5p were screened and identified. Firstly, we monitored that the expression of KIF23 was significantly enhanced in PCa tissues compared with that in normal tissues at both mRNA and protein levels (Fig. 4a, b). As well, the expression of KIF23 was notably elevated in PCa cell lines compared with that in normal cell line at both mRNA and protein levels (Fig. 4c, d). Spearman’s correlation coefficient indicated that miR-15a-5p expression was negatively correlated with KIF23 expression (Fig. 4e). Bioinformatics tool starBase v2.0 concluded that a special binding site existed between miR-15a-5p and KIF23 3′ UTR (Fig. 4f). Dual-luciferase reporter assay presented that miR-15a-5p mimic transfection substantially reduced the luciferase activity in 293T cells transfected with KIF23 3′ UTR-WT compared with that in 293T cells transfected with KIF23 3′ UTR-MUT, and RNA pull-down assay presented that Bio-miR-15a-5p largely enriched the abundance of KIF23 in the beads (Fig. 4g). Furthermore, we observed that the expression of KIF23 plummeted with the enrichment of miR-15a-5p at both mRNA and protein levels (Fig. 4h, i). These data summarized that miR-15a-5p interacted with KIF23 and modulated the expression of KIF23.

### The impacts of KIF23 knockdown in PCa cells were inhibited by miR-15a-5p inhibitor

**Fig. 5:**
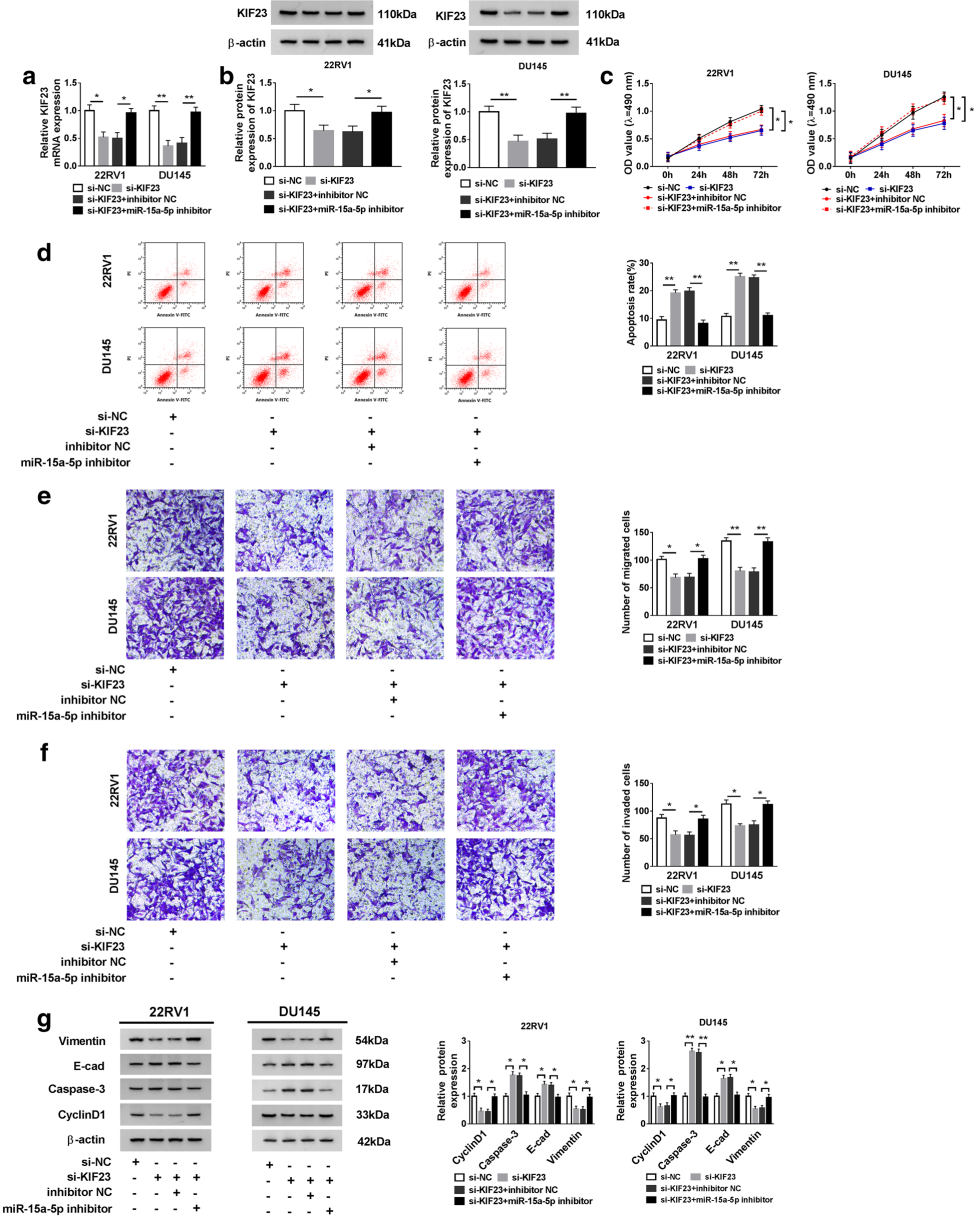
Inhibition of KIF23 blocked the function of KIF23 knockdown in PCa cells. 22RV1 and DU145 cells were introduced with si-KIF23, si-NC, si-KIF23 + miR-15a-5p inhibitor and si-KIF23 + inhibitor NC, respectively. **a**, **b** The expression of KIF23, **c** cell proliferation, **d** cell apoptosis, **e** cell migration (×100), **f** cell invasion (×100) and **g** the levels of Vimentin, E-cad, Caspase-3 and CyclinD1 were detected. *P < 0.05, **P < 0.01

To address the effects of the interaction between miR-15a-5p and KIF23, 22RV1 and DU145 cells were introduced with si-KIF23 and si-KIF23 + miR-15a-5p inhibitor, respectively, si-NC and si-KIF23 + inhibitor NC as the control. Firstly, the expression of KIF23 was detected, and the result showed that KIF23 was remarkably down-regulated in cells transfected with si-KIF23 but thereupon up-regulated with the transfection of si-KIF23 + miR-15a-5p inhibitor at both mRNA and protein levels (Fig. 5a, b). Then, the ability of cell proliferation was inhibited by si-KIF23 but recovered by si-KIF23 + miR-15a-5p inhibitor (Fig. 5c). The apoptosis rate of 22RV1 and DU145 cells was stimulated by KIF23 knockdown but blocked by miR-15a-5p inhibition (Fig. 5d). The number of migrated and invaded cells was decreased by the transfection of si-KIF23 but reincreased by the transfection of si-KIF23 + miR-15a-5p inhibitor (Fig. 5e, f). Additionally, the levels of Vimentin and CyclinD1 were reduced in cells transfected with si-KIF23 but enriched in cells transfected with si-KIF23 + miR-15a-5p inhibitor, while the levels of E-cad and Caspase-3 were on the contrary (Fig. 5g). Above data implied that miR-15a-5p inhibition could abolish the role of KIF23 knockdown.

### PVT1 regulated the expression of KIF23 through miR-15a-5p

**Fig. 6:**
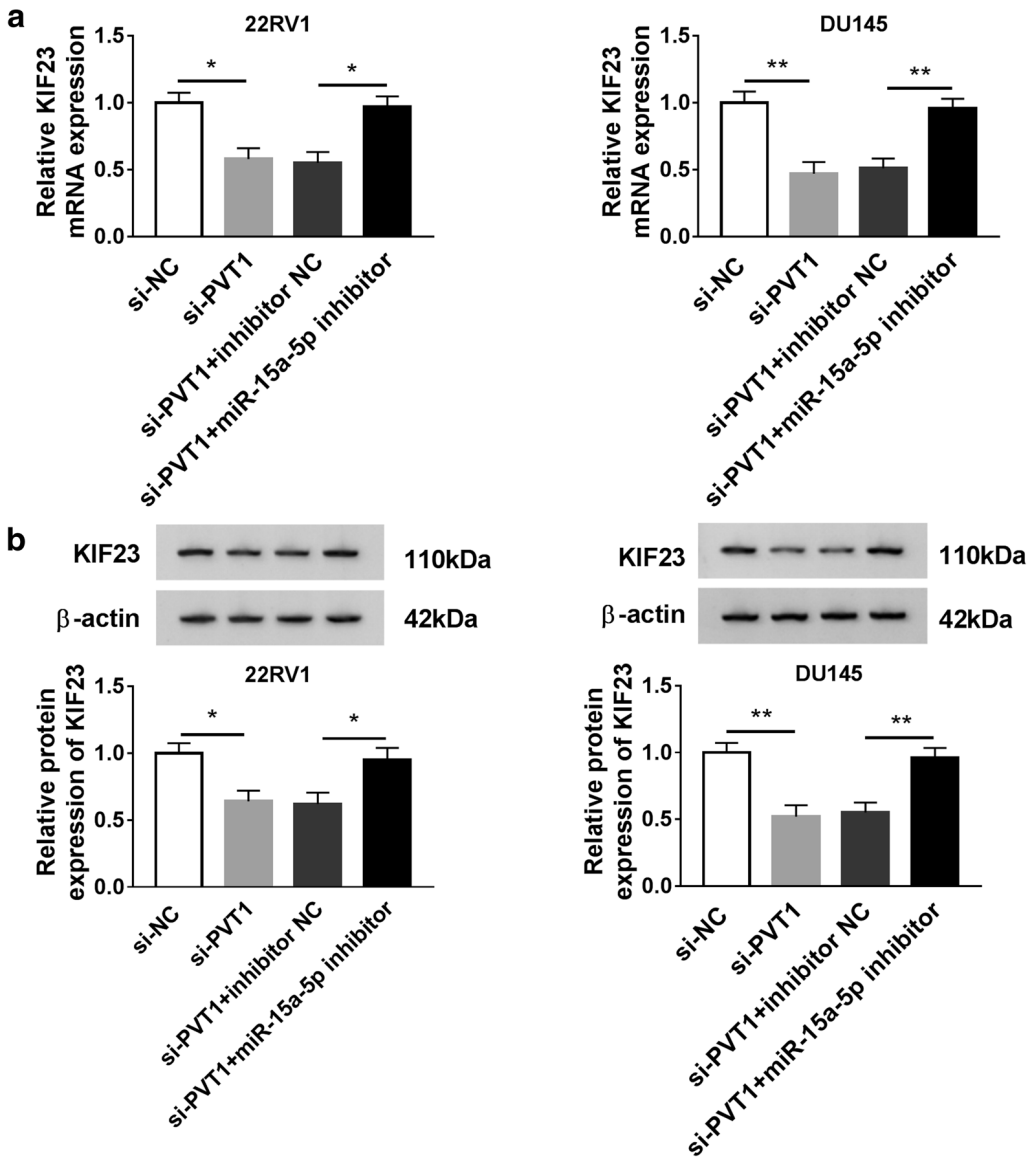
The expression of KIF23 was regulated by PVT1 through miR-15a-5p in PCa cells. **a**, **b** The expression of KIF23 at mRNA and protein levels was checked in 22RV1 and DU145 cells transfected with si-PVT1 and si-PVT1 + miR-15a-5p inhibitor, respectively, si-NC or si-PVT1 + inhibitor NC as the control. *P < 0.05, **P < 0.01

To further investigate whether PVT1 regulated KIF23 through miR-15a-5p, 22RV1 and DU145 cells were inserted with si-PVT1 and si-PVT1 + miR-15a-5p inhibitor, respectively, si-NC and si-PVT1 + inhibitor NC as the control. The consequence of qRT-PCR and western blot displayed that the expression of KIF23 depleted by si-PVT1 was restored by si-PVT1 + miR-15a-5p inhibitor compared to corresponding control at both mRNA and protein levels (Fig. 6a, b). Furthermore, rescue experiments for KIF23 overexpression rescuing the effects of PVT1 knockdown were shown in Additional file 3: Figure S2. The data confirmed that PVT1 exerted its role in PCa progression by inducing KIF23 through mediating miR-15a-5p.

### PVT1 knockdown impeded tumor progression in vivo

**Fig. 7:**
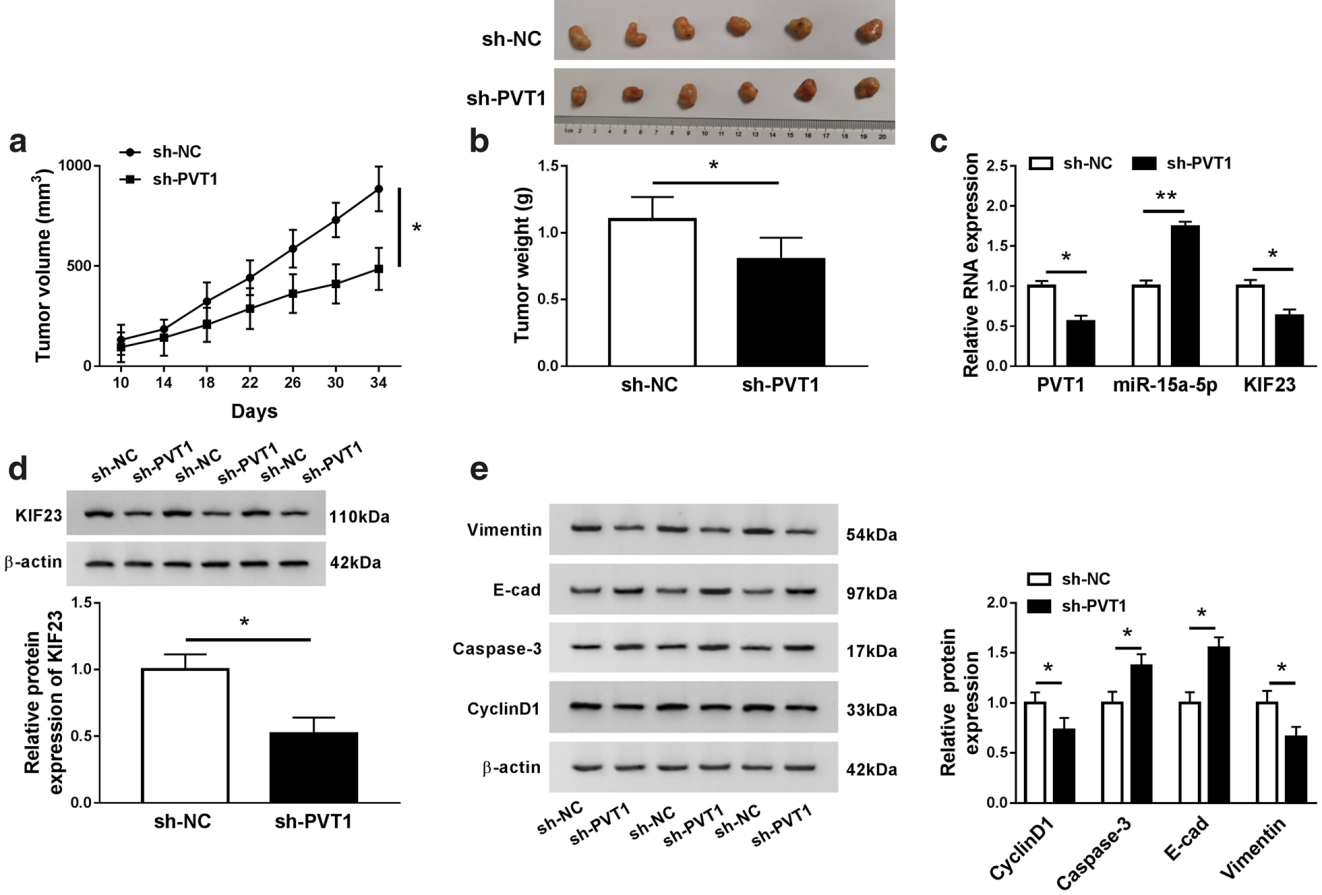
Knockdown of PVT1 impeded the PCa progression in vivo. **a** The tumor volume was calculated every 4 day after 10 day-inoculation. **b** The tumor weight was measured after 34 days. **c** The expression of PVT1, miR-15a-5p and KIF23, **d** the expression of KIF23 at the protein levels, **e** and the levels of Vimentin, E-cad, Caspase-3 and CyclinD1 were examined in removed tumor tissues using qRT-PCR or western blot. *P < 0.05, **P < 0.01

To examine the role of PVT1 on tumorigenesis in vivo, DU145 cells with stable PVT1 knockdown were inoculated into the subcutaneous tissues of nude mice. The efficiency of PVT1 knockdown in DU145 cells transfected with sh-PVT1 was displayed in Additional file 4: Figure S3, the data showed that the expression of PVT1 was remarkably declined in DU145 cells transfected with sh-PVT1 compared to sh-NC. The tumor volume, recorded every 4 days at 10 days post-injection, was obviously decreased in sh-PVT1-injected groups relative to sh-NC (Fig. 7a). The tumor weight was measured at the end, and the data indicated that the tumor weight was weaker in sh-PVT1-injected groups relative to sh-NC groups (Fig. 7b). Whereafter, the expression of PVT1, miR-15a-5p and KIF23 was detected in removed tumor tissues, and we found the expression of PVT1 and KIF23 was significantly declined, while miR-15a-5p expression was strengthened by qRT-PCR analysis (Fig. 7c). The expression of KIF23 at the protein level was consistent with its mRNA level (Fig. 7d). Moreover, western blot analysis manifested that the levels of Vimentin and CyclinD1 were markedly weakened in sh-PVT1 groups compared with that in sh-NC groups, while the levels of E-cad and Caspase-3 were reinforced in sh-PVT1 groups relative to that in sh-NC groups (Fig. 7e). These data ascertained that PVT1 knockdown blocked tumorigenesis and progression through the downregulation of KIF23 and the upregulation of miR-15a-5p in vivo.

## Discussion

PCa accounts for 19% of all new cancer cases and 9% of all cancer-related deaths, according to the cancer statistics report [26]. The overall survival rate for PCa patients is unsatisfactory within 5 years. Hence, the identification of novel mechanisms and therapeutic biomarkers is needed for the improvement of PCa. In this study, we observed that PVT1 was aberrantly up-regulated in PCa, and PVT1 downregulation inhibited the malignant phenotypes of PCa cells in vitro and in vivo. The targeted relationship between miR-15a-5p and PVT1 or KIF23 was validated, and we found that PVT1 regulated the expression of KIF23 through miR-15a-5p, suggesting the regulatory effects of PVT1/miR-15a-5p/KIF23 axis in the progression of PCa.

The research of lncRNAs in RNA biology is noticeable, and the exploration of certain lncRNAs in PCa leads us to determine the role of them as diagnostic, treatment and prognostic biomarkers [27]. Several previous studies mentioned the partial functions of PVT1 in PCa. For example, PVT1 knockdown blocked the proliferation and metastasis of PCa cells, partly via reducing the phosphorylated role of p38 [28]. PVT1 contributed to migration and invasion of PCa cells, which attributed to the activation of epithelial-mesenchymal transition (EMT) by the regulation of miR-186 and Twist1 [29]. PVT1 was up-regulated in PCa, and PVT1 regulated cell viability and apoptosis through the modulation of miR-146a [30]. These findings indicated that the action mechanisms of PVT1 in PCa were complex and diverse. Consistent with these studies, PVT1 was also highly expressed in PCa tissues and cells in our research. Besides, functional analyses deemed that interference of PVT1 ameliorated the malignant activities of PCa cells, suggesting the carcinogenic effect of PVT1.

To further understand the manner of PVT1 action, we attempted to seek for a novel mechanism of PVT1 in the progression of PCa. Through the prediction of bioinformatics and the verification of dual-luciferase reporter assay, miR-15a-5p was identified as a target of PVT1. Previous studies concluded that miR-15a was down-regulated in PCa tissues and cells, and its enrichment could suppress invasion and proliferation of PCa cells [31, 32]. In combination with the performance of miR-15a-5p in other cancers [19, 20, 33], we deduced that miR-15a-5p might act as a tumor suppressor to inhibit the malignant behaviors of PCa. In agreement with these studies, we observed that the expression of miR-15a-5p was decreased in PCa, and its inhibition could eliminate the effects of PVT1 interference in PCa cells. Interestingly, miR-15a-5p was proved as an oncogene in certain cancers, including cervical cancer [34] and colorectal adenocarcinoma [35]. This might be due to the different expression patterns of miR-15a-5p in diverse cancer types.

KIF23 was screened and confirmed as a direct target of miR-15a-5p. KIF23 was reported to be up-regulated in numerous cancers, leading to the accelerative cell proliferation, migration, poor prognostic and other harmful acts [23, 24, 36]. In PCa, the involvement of other members of the kinesin family in PCa had been expounded, such as KIF11, KIFC1, and KIF4A [37–39]. All of them were overexpressed in PCa and predicted poor prognosis. Consistent with the above findings, KIF23 was also highly expressed in PCa tissues and cells through qRT-PCR and western blot analyses. Besides, KIF23 knockdown attenuated proliferation, migration and invasion but promoted apoptosis of PCa cells, implying that KIF23 was a tumor promoter in PCa.

## Conclusion

Collectively, the expression of PVT1 and KIF23 was reinforced in PCa, while the expression of miR-15a-5p was declined. Knockdown of PVT1 suppressed the progression of PCa both in vitro and in vivo by mediating the miR-15a-5p/KIF23 network. Our present study provided a novel mechanism of PCa progression and broadened horizons to PVT1 as a biomarker for the treatment of PCa.

## Supplementary information

**Additional file 1: Table S1.** Correlation between PVT1 expression and clinicopathological parameters of patients.

**Additional file 2: Figure S1.** The predicted target miRNAs of PVT1 and their expression in PCa tissues and si-PVT1-transfected PCa cells. (A) The potential binding site between PVT1 and target miRNAs were analyzed by Starbase. (B) The expression of target miRNAs, including miR-515-5p, miR-24-3p, miR-512-3p, miR-15a-5p, miR-21-5p and miR-17-5p, was detected using qRT-PCR in PCa tissues and normal tissues. (C) The expression of miR-515-5p, miR-24-3p, miR-512-3p and miR-15a-5p in 22RV1 and DU145 cells transfected with si-PVT1 or si-NC was detected by qRT-PCR. *P < 0.05.

**Additional file 3: Figure S2.** KIF23 overexpression rescued the effects of PVT1 knockdown. 22RV1 and DU145 cells were transfected with si-PVT1 or si-VT1 + oe-KIF23, with si-NC or or si-PVT1 + vector as the control. (A andB) The expression of KIF23 in these transfected cells was detected by qRT-PCR an western blot. (C) Cell proliferation was assessed by MTT assay. (D) Cell apoptosis was monitored by flow cytometry assay. (E and F) Cell migration and cell invasion in these transfected cells were investigated by transwell assay. (G) The expression of Vimentin, E-cad, Caspase-3 and CyclinD1 was quantified by western blot in these transfected cells. *P < 0.05, **P < 0.01.

**Additional file 4: Figure S3.** The expression of PVT1 in DU145 cells transfected with sh-PVT1 was notably declined. (A) The expression of PVT1 in DU145 cells transfected with sh-PVT1 or sh-NC was measured using qRT-PCR. *P < 0.05.

## Data Availability

The analyzed data sets generated during the present study are available from the corresponding author on reasonable request.

## Abbreviations

PCa: Prostate cancer
lncRNAs: Long non-coding RNAs
miR-15a-5p: MicroRNA-15a-5p
KIF23: Kinesin family member 23
qRT-PCR: Quantitative real-time polymerase chain reaction
EMT: Epithelial–mesenchymal transition
RPMI: Roswell Park Memorial Institute 1640
FBS: Fetal bovine serum
DMEM: Dulbecco’s Modified Eagle Medium
