# Supplementary material for: Knockdown of lncRNA PVT1 inhibits prostate cancer progression in vitro and in vivo by the suppression of KIF23 through stimulating miR-15a-5p
Source: Cancer Cell Int. 2020 Jul 2;20:283. doi: 10.1186/s12935-020-01363-z (PMC7330980; doi:10.1186/s12935-020-01363-z)
Supplement: Supplementary file 1 — Additional file 1: Table S1. Correlation between PVT1 expression and clinicopathological parameters of patients. [file 12935_2020_1363_MOESM1_ESM.doc]

Table S1 Correlation between PVT1 expression and clinicopathological parameters of patients with PCa

| Clinicopathological characteristics | Total | PVT1 low  expression(n=13) | PVT1 high  expression(n=12) | P value |  |
| --- | --- | --- | --- | --- | --- |
| Age |  |  |  |  |  |
| <60 | 10 | 4 | 6 | 0.4283 |  |
| ≥60 | 15 | 9 | 6 |  |  |
|  |  |  |  |  |  |
| Gleason score |  |  |  |  |  |
| <7 | 12 | 10 | 2 | 0.0048 | * |
| ≥7 | 13 | 3 | 10 |  |  |
|  |  |  |  |  |  |
| Tumor stage |  |  |  |  |  |
| T1-T2 | 16 | 11 | 5 | 0.0414 | * |
| T3-T4 | 9 | 2 | 7 |  |  |
|  |  |  |  |  |  |
| Lymph node metastasis |  |  |  |  |  |
| N0 | 14 | 10 | 4 | 0.0472 | * |
| N1 | 11 | 3 | 8 |  |  |
|  |  |  |  |  |  |
| Distant metastasis |  |  |  |  |  |
| M0 | 17 | 12 | 5 | 0.0112 | * |
| M1 | 8 | 1 | 7 |  |  |

“*”: statistically significant
