# Supplementary figures and images for: Knockdown of lncRNA PVT1 inhibits prostate cancer progression in vitro and in vivo by the suppression of KIF23 through stimulating miR-15a-5p
Source: Cancer Cell Int. 2020 Jul 2;20:283. doi: 10.1186/s12935-020-01363-z (PMC7330980; doi:10.1186/s12935-020-01363-z)

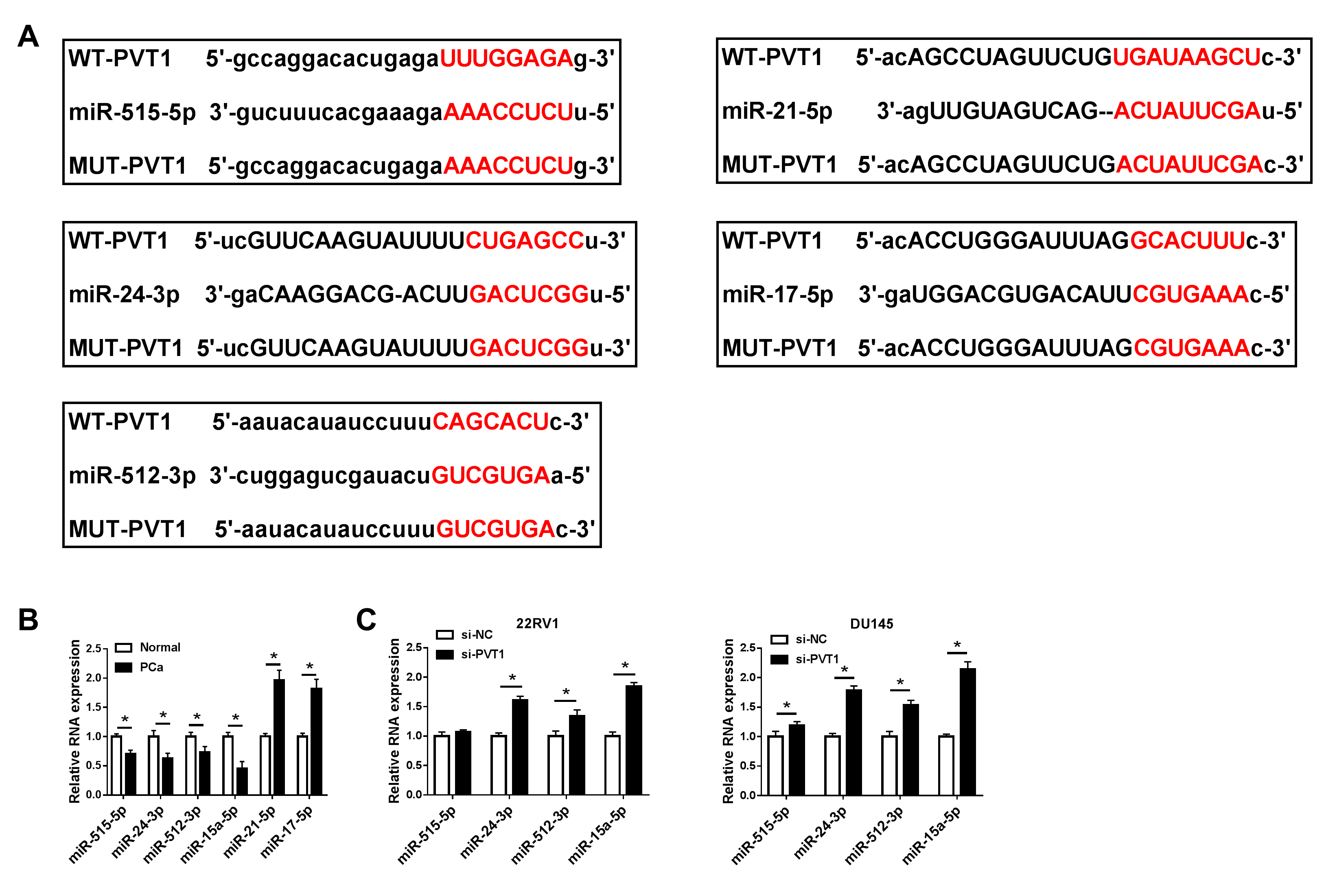

Supplement: Supplementary file 2 — Additional file 2: Figure S1. The predicted target miRNAs of PVT1 and their expression in PCa tissues and si-PVT1-transfected PCa cells. (A) The potential binding site between PVT1 and target miRNAs were analyzed by Starbase. (B) The expression of target miRNAs, including miR-515-5p, miR-24-3p, miR-512-3p, miR-15a-5p, miR-21-5p and miR-17-5p, was detected using qRT-PCR in PCa tissues and normal tissues. (C) The expression of miR-515-5p, miR-24-3p, miR-512-3p and miR-15a-5p in 22RV1 and DU145 cells transfected with si-PVT1 or si-NC was detected by qRT-PCR. *P < 0.05. [file 12935_2020_1363_MOESM2_ESM.tif]

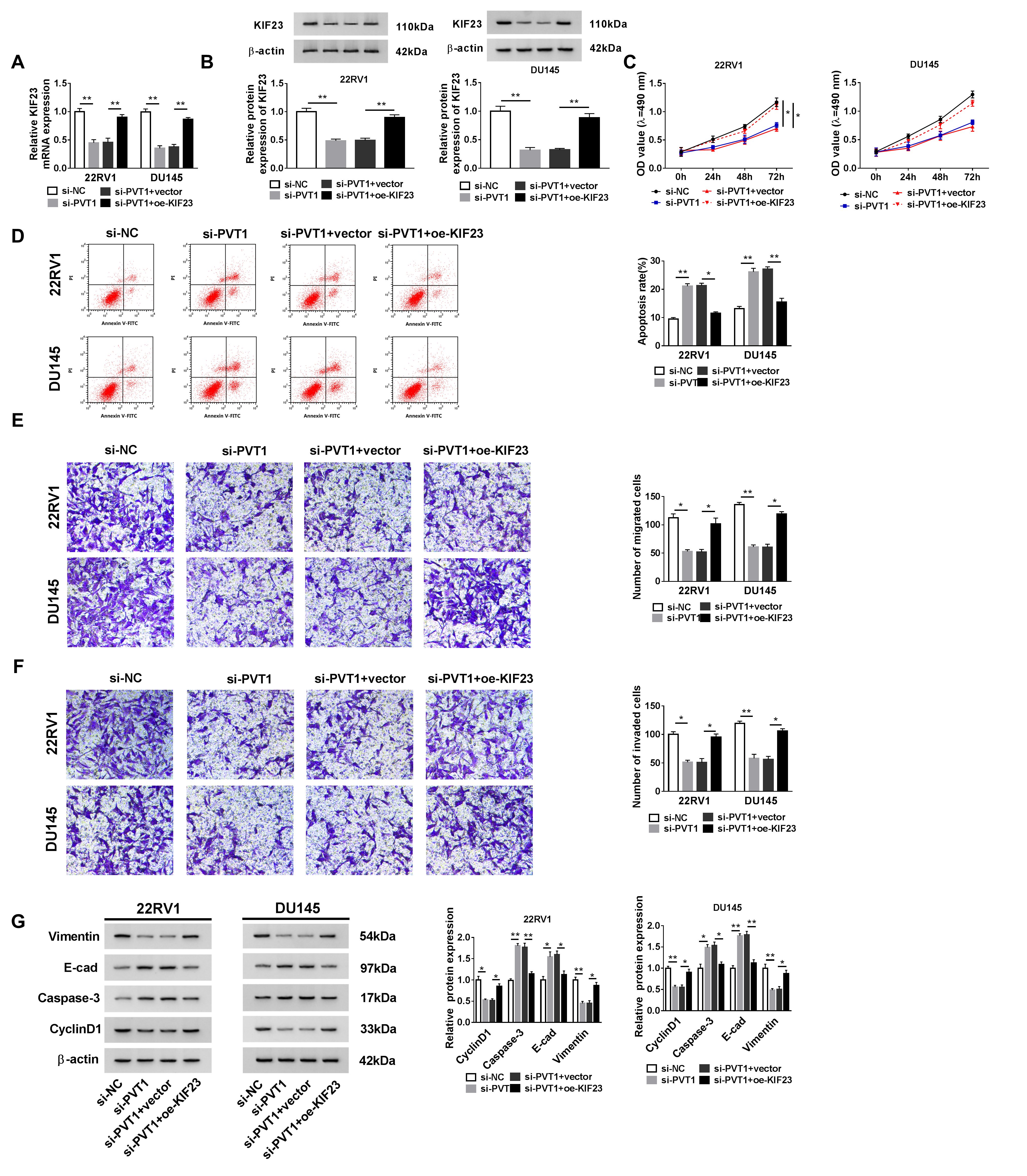

Supplement: Supplementary file 3 — Additional file 3: Figure S2. KIF23 overexpression rescued the effects of PVT1 knockdown. 22RV1 and DU145 cells were transfected with si-PVT1 or si-VT1 + oe-KIF23, with si-NC or or si-PVT1 + vector as the control. (A andB) The expression of KIF23 in these transfected cells was detected by qRT-PCR an western blot. (C) Cell proliferation was assessed by MTT assay. (D) Cell apoptosis was monitored by flow cytometry assay. (E and F) Cell migration and cell invasion in these transfected cells were investigated by transwell assay. (G) The expression of Vimentin, E-cad, Caspase-3 and CyclinD1 was quantified by western blot in these transfected cells. *P < 0.05, **P < 0.01. [file 12935_2020_1363_MOESM3_ESM.tif]

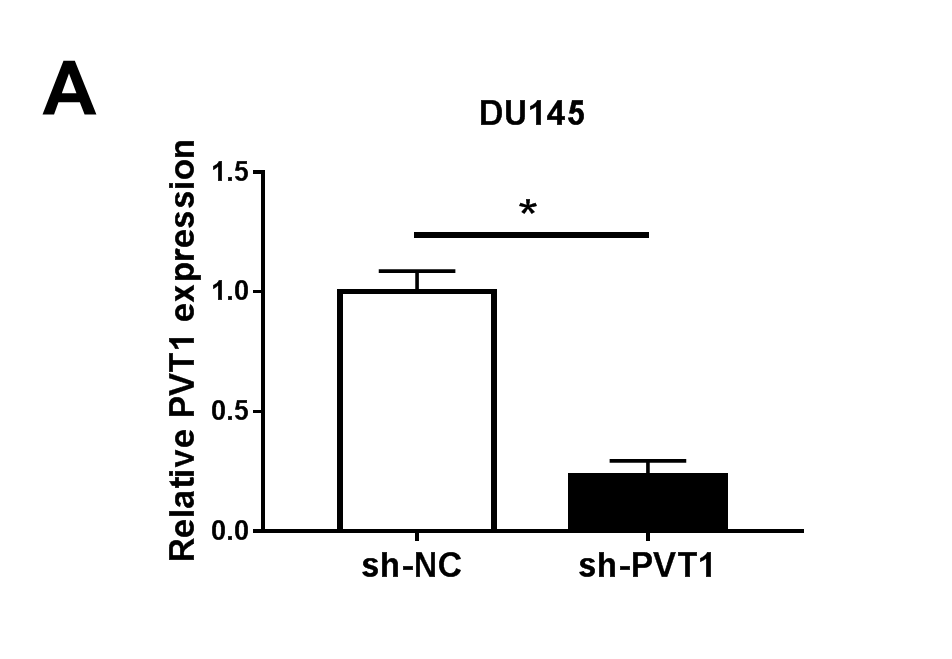

Supplement: Supplementary file 4 — Additional file 4: Figure S3. The expression of PVT1 in DU145 cells transfected with sh-PVT1 was notably declined. (A) The expression of PVT1 in DU145 cells transfected with sh-PVT1 or sh-NC was measured using qRT-PCR. *P < 0.05. [file 12935_2020_1363_MOESM4_ESM.tif]
